# Pulaski County (Ind.) Medical Society

**Published:** 1867

**Authors:** F. B. Thomas, I. B. Washburn

**Affiliations:** Pres’t.; Sec’y.


### PULASKI COUNTY (IND.) MEDICAL SOCIETY.

Pursuant to a call of the physicians of Pulaski and adjoining counties, the following gentlemen assembled in Winamac, Ind., April 2d, 1867, for the purpose of organizing a Medical Society:—F. B. and A. McD. Thomas, A. M. Pearson, James Tolerton, H. Kittinger, Winamac; J. W. C. Eaton, and N. S. Hazen, Pulaski; C. G. Hartman, Francisville; James H. Smith, Harrison Township; I. B. Washburn and J. B. Moore, (student,) Star City; L. D. Glazebrook, San Pierre, Stark Co; and James Thomas, Royal Centre, Cass Co.

On motion, F. B. Thomas was chosen Chairman, and I. B. Washburn Secretary.

On motion of Dr. Jas. Thomas, a committee of five was appointed to draft a constitution and by-laws. The Chairman appointed Drs. Glazebrook, Hartman, Pearson, Tolerton, and A. Thomas. The committee reported a constitution and by-laws in the usual form, which were adopted.

After the adoption of the constitution, the Society proceeded to elect officers, which resulted as follows:—*President*, F. B. Thomas; *Secretary*, I. B. Washburn; *Treasurer*, A. M. Pearson; *Censors*, L. D. Glazebrook, James Tolerton, and A. McD. Thomas.

The Society adopted a fee bill, equalizing the charges for professional services, and harmonizing with those of neighboring societies.

Dr. Washburn offered the following resolutions, which were unanimously adopted:—

*Whereas*, From the teaching and practice of a certain class of *self-styled* physicians, the unnatural crime of procuring abortion or miscarriage, for the purpose of gratifying the lust, ambition, or caprice of those who do not wish to bear maternal responsibilities; therefore,

*Resolved*, First, That members of this Society will in no case countenance the practice, except to save life, and then only after consultation with a physician of good standing, when such consultation is possible.

*Resolved*, Secondly, That members of this Society who may be cognizant of the procuring of criminal abortion, apprise the Grand Juries of their respective districts of the facts in the case, looking to a speedy prosecution of the same.

On motion, Dr. L. D. Glazebrook was appointed on essay, and of the proceedings of the Society for publication in the CHICAGO MEDICAL JOURNAL, *Chicago Medical Examiner*, and *Cincinnati Lancet & Observer*.

On motion, adjourned, to meet in Winamac, the second Tuesday in June, 1867. F. B. THOMAS, *Pres't.*

I. B. WASHBURN, *Sec'y.*

